# Multi-metal 4D printing with a desktop electrochemical 3D printer

**DOI:** 10.1038/s41598-019-40774-5

**Published:** 2019-03-08

**Authors:** Xiaolong Chen, Xinhua Liu, Mengzheng Ouyang, Jingyi Chen, Oluwadamilola Taiwo, Yuhua Xia, Peter R. N. Childs, Nigel P. Brandon, Billy Wu

**Affiliations:** 1 Dyson School of Design Engineering, Imperial College London, London, UK; 2 Department of Earth Science and Engineering, Imperial College London, London, UK; 3 Department of Materials, Imperial College London, London, UK

## Abstract

4D printing has the potential to create complex 3D geometries which are able to react to environmental stimuli opening new design possibilities. However, the vast majority of 4D printing approaches use polymer based materials, which limits the operational temperature. Here, we present a novel multi-metal electrochemical 3D printer which is able to fabricate bimetallic geometries and through the selective deposition of different metals, temperature responsive behaviour can thus be programmed into the printed structure. The concept is demonstrated through a meniscus confined electrochemical 3D printing approach with a multi-print head design with nickel and copper used as exemplar systems but this is transferable to other deposition solutions. Improvements in deposition speed (34% (Cu)–85% (Ni)) are demonstrated with an electrospun nanofibre nib compared to a sponge based approach as the medium for providing hydrostatic back pressure to balance surface tension in order to form a electrolyte meniscus stable. Scanning electron microscopy, X-ray computed tomography and energy dispersive X-ray spectroscopy shows that bimetallic structures with a tightly bound interface can be created, however convex cross sections are created due to uneven current density. Analysis of the thermo-mechanical properties of the printed strips shows that mechanical deformations can be generated in Cu-Ni strips at temperatures up to 300 °C which is due to the thermal expansion coefficient mismatch generating internal stresses in the printed structures. Electrical conductivity measurements show that the bimetallic structures have a conductivity between those of nanocrystalline copper (5.41 × 10^6^ S.m^−1^) and nickel (8.2 × 10^5^ S.m^−1^). The potential of this novel low-cost multi-metal 3D printing approach is demonstrated with the thermal actuation of an electrical circuit and a range of self-assembling structures.

## Introduction

Additive manufacturing (AM), which is also commonly known as 3D printing, fabricates complex 3D geometries by sequentially joining material together layer-by-layer^1^. Due to the design flexibility that AM offers, there has been considerable industrial uptake in fields such as: aerospace^2^, automotive^3^, medical^4–6^ and energy^7–10^ to name but a few. Early applications of AM focused on the use of polymers due to the ease of consolidation, either through a photo-polymerisation (stereolithography)^11^ or thermal process (fused deposition modelling (FDM))^12^. However, there has been increased uptake of metal based AM as the technology transitions from being primarily used as a prototyping tool to making end products^1^. Here, the main technologies include: direct metal laser sintering^13^, electron beam melting^14^, directed energy deposition^15^ and FDM using a metal filled polymer filament^16^. In the vast majority of these cases only a single material is used however there are current research efforts to expand metal 3D printing to include multi-material capabilities. For example, Li *et al*.^17^ demonstrated the deposition of various metal carbides, such as titanium carbide, tungsten carbide, and silicon carbide into a titanium alloy matrix via a process whereby titanium wires and the metal carbide powder are fed into a melt pool formed by a laser. The wire and powder fed process makes metal composite printing feasible however, the printed part has limited dimensional accuracy and high surface roughness.

Onuike *et al*.^18^ demonstrated the fabrication of a copper and nickel alloy bimetallic structure using laser engineering net shaping (LENS), whereby a metallic powder is fed into a melt pool formed with a laser. Here they explored bimetallic structures through a sequential deposition and a functional grading of the two alloys with both approaches showing improvements in the thermal conductivity relative to a baseline Inconel 718 alloy. However, due to the powder spraying based approach the dimensional accuracy of the printed parts were limited and also the aggressive thermal conditions resulted in significant thermal stresses which can lead to layer delamination in multi-metal systems^19^.

Other techniques including LENS coating^20^, hybrid metal additive manufacturing^21^, robocasting^22^ and metal jetting based approaches^23^ have been investigated for the fabrication of metal-based multi-material 3D printing. However, one of the main drawbacks of these techniques is the high capital cost of the equipment and associated safety risks due to the use of high power lasers, metal powders and/or the need for high temperature heat treatments. Electrochemical additive manufacturing (ECAM) is a relatively new form of metal AM which uses the localised electrochemical deposition of metal ions from electrolyte solutions to create metal structures^24–27^. The advantage here is that thermal processes are not needed, which enables the system to be lower cost and safer, however challenges around the deposition speed still need to be overcome.

In the vast majority of AM applications, uses have focused on printing with only a single material, however there is increasing interest in multi-material 3D printing which can provide a larger range of functionalities^28,29^. One of the new design possibilities includes 4D printing techniques which have been investigated as a means of creating self-assembling and self-regulating structures that change shape due to environmental stimuli such as temperature, humidity or light^30–37^.

A common method of creating 4D structures is to synthesize active materials with temperature-responsive properties and to control the thermal boundary conditions to achieve different temporary shapes. Ge *et al*.^38^ for instance, proposed a method of adding shape memory polymer (SMP) fibres into an elastomeric matrix to fabricate a hinge. By utilizing the thermal responsive properties of SMPs and the different glass transition temperatures of the materials, the hinge bent with a maximum deformation angle of ~20°. Wu *et al*.^39^ and Yu *et al*.^40^ then progressed this concept by adding different types of SMP fibres with different glass transition temperatures. With this modification, the printed hinges exhibited more varied movements however the maximum operating temperature was limited by the thermal stability of the polymers used, with this often below 100 °C due to the use of a water bath to control the environmental temperature.

These examples, however, only demonstrate limited motions and at modest operational temperatures. To improve this, Zhang *et al*.^41^ fabricated a six-petal leaf with a bilayer structure (polylatic acid on paper). By changing the external temperature, the bilayer could uniformly curl into a flower shape. This technique is able to create different kinds of complex structures such as helical structures and corrugated structures. However, the bilayer is fragile with delamination possible after repeated thermal cycles.

Apart from SMP fibre composite structures, Bakarich *et al*.^42^ fabricated a 4D valve with hydrogels. Due to the temperature-sensitive properties of their developed hydrogel, the designed valve was able to control the environmental temperature (20 °C–60 °C) of the working fluid through moderating the flow rate. However, thermal stability beyond this range remains a challenge.

Another approach to 4D printing is to use humidity-responsive materials to actuate the transformation upon absorbing or releasing moisture^43,44^. Raviv *et al*.^32^ printed objects with a rigid plastic base and humidity-responsive materials which expand upon moisture changes. The volume of printed object is able to extend and fold by as much as 200% relative to the original size. However, this expansion is fragile, with the repeatability and reversibility of the folding and unfolding motion limited when more cycles are applied. Zhang *et al*.^43^ also fabricated composite films which are able to quickly transform in response to humidity changes. Moreover, shape changes such as bending, helical twisting and rolling, can be driven not only by changes in humidity but also by ultraviolet light due to the photomechanical response of the material.

Besides temperature-actuated and humidity-actuated materials, current-actuated materials have also been explored as potential materials for 4D printing. Okuzaki *et al*.^45^ fabricated a bent accordion-like object by using a conductive polymer combination of polyaniline and polypyrrole (PPy). After applying an electric potential, the shape transformed due to the water vapour in the PPy evaporating due to Joule heating. However, the reversibility of this movement is slow due to the sluggish rehydration of the polymer. Another strategy for the fabrication of electrically responsive materials includes adding conductive media into SMPs. For instance, Lu *et al*.^46^ combined carbon nanotubes with a shape-memory polymer. When applying an electrical current the film quickly folded from a flat shape to ‘U’-like temporary shape with the original shape recovered within 120 s. Some other active materials, such as magnetic-responsive materials^47,48^, light-responsive materials^49,50^, and pH-responsive material^51^ have also been explored as materials for 4D printing.

In summary, there is an increased interest in 4D printing to create passive structures that respond to stimuli such as temperature, humidity and current flow. In the majority of these cases, these are composite polymer based materials which allows for ease of fabrication but limits their maximum operating temperature and transient performance due to poor thermal stability and conductivity. In some cases, the printed objects also exhibit anisotropic mechanical properties due to the poor inter-layer adhesion^52^ and some of the constructs also have poor durability due to the mechanical degradation during thermo-mechanical cycling^53^. Metals have a much higher melting temperature than polymers and thus there is potential in creating 4D structures with high operating temperatures and mechanical robustness. However, creating a low-cost multi-material metal printer has not been achieved to-date. Current multi-metal 3D printing approaches are nearly all thermally based approaches where a blown powder or wire is fed into a melt pool formed by a laser within an inert atmosphere. Compositional grading/multi-material capabilities can be achieved by varying the powder/wire however the resolution and cost of these processes are poor with significant thermal stresses resulting in low print quality. This work, thus presents a novel ECAM based approach for creating multi-metal structures with high resolution and low cost which opens the possibility for creating complex high temperature 4D self-assembling/actuating structures using inexpensive components and materials. Examples of the merits of this approach are demonstrated through the fabrication of copper-nickel bimetallic strips which are able to achieve programmable mechanical responses to thermal stimuli.

## Results and Discussions

Here, we demonstrate a novel approach to achieve 4D metal structures through multi-metal ECAM using a meniscus confined approach with multiple syringes. This work is built upon previous work by Chen *et al*.^24^ who developed a low-cost desktop electrochemical 3D printer. In the paper, we report a systematic review of factors, such as layer thickness, temperatures, deposition positions which influence the dynamic motion of printed copper-nickel bimetallic strips. Further analysis into the metallic properties of the structures, such as, electrical conductivity and surface morphology is presented through scanning electron microscopy (SEM) and X-ray computed tomography (XCT).

### Rig design for the multi-metal electrochemical 3D printer

**Figure 1:**
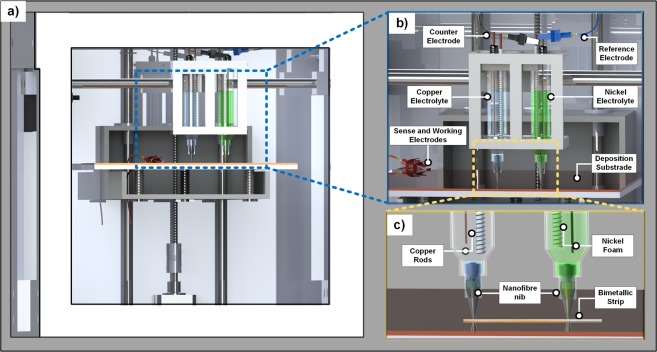
Illustration of the low cost electrochemical multi-metal 3D printer. (**a**) Front view. (**b**) Print head setup. (**c**) Detailed view highlighting the deposition nozzles and the deposited bimetallic strip.

Figure 1a–c presents a schematic of the low cost electrochemical multi-metal 3D printer which was converted from a commercial FDM 3D printer. The modified rig consists of a stage with two in-line plastic syringes separated by a distance of 80 mm. One syringe contains a copper sulphate electrolyte (blue solution) and one holds a nickel sulphate electrolyte solution (green solution). For the copper sulphate syringe assembly, two copper wires were inserted into the electrolyte, one as a counter electrode and one as a reference electrode. To maintain an accurate distance between two electrodes, the reference electrode was connected to an insulated holder. The counter electrode wire then wraps around the insulated rod which increases the surface area. Sense and working electrode probes from a potentiostat are attached to a copper substrate platform where the deposition occurs. The setup for the syringe containing the nickel solution was similar, but the materials serving as the reference and counter electrodes were changed from copper wires to nickel foams. The deposition unit has two movements (X and Y axis) and the deposition platform is able to move along Z axis. The movements are computer controlled by stepper motors.

### Fabrication and characterisation of printed bimetallic strips

**Figure 2:**
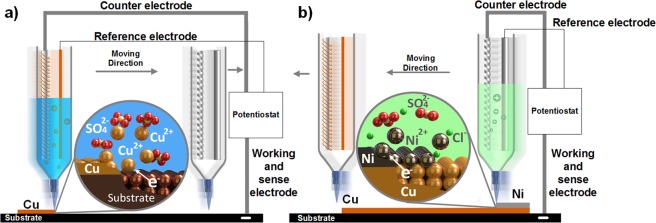
Schematic illustration of the multi-material 3D printing process. (**a**) The meniscus confined copper electrodeposition process. (**b**) The meniscus confined nickel electrodeposition process.

The fabrication process for a copper-nickel bimetallic strip is schematically demonstrated in Fig. 2. Here one syringe is filled with electrolyte for deposition while the other one remains empty to avoid undesirable electrolyte mixing solutions. In the first stage, a copper layer is deposited from an aqueous copper sulphate electrolyte (Fig. 2a). Here, a stable electrolyte meniscus is formed between the nozzle and the substrate by balancing the hydraulic head of the electrolyte with the surface tension of the electrolyte and back pressure of a porous media in the print nozzle. A potentiostat then applies a constant potential to reduce the Cu^2+^ ions in the electrolyte to metallic copper on the substrate. Simultaneously, the consumed Cu^2+^ ions are replenished from counter electrode through the oxidation of the electrode, maintaining the overall concentration but forming a concentration gradient in the solution. This concentration gradient can affect the current density and morphology of the printed copper. Previously, Chen *et al*.^24^ used sponges with a random open pore structure to provide suitable back pressure to form a stable meniscus, however, in this work an electrospun nanofibre nib was used. This has the advantage of providing suitable back pressure against the hydraulic head whilst minimising diffusion resistance. This is exemplified by a 34% increase in the current density (from 480 mA.cm^−2^ to 640 mA.cm^−2^) when depositing copper at 5 V vs Cu with a 1 M copper sulphate solution. The position of the deposition is controlled by the movement of the printing head.

After the copper layer was deposited, the copper electrolyte was removed and a nickel layer was deposited on top of this as illustrated in Fig. 2b. Here the set-up was the same but with nickel counter and reference electrodes, as well as a nickel based electrolyte. A potentiostat then applied a constant potential again to reduce the Ni^2+^ ions in the electrolyte to metallic nickel on top of the deposited copper layer. The print head then moves on the path defined, depositing nickel where the meniscus bridge and potential is applied, with repeated passes increasing the layer thickness. The use of the nanofibre nib over the sponge approach was highlighted by a current density increase of 85% (from 100 mA.cm^−2^ to 190 mA.cm^−2^) at a deposition potential of 2 V vs Ni. SEM micrographs and optical images of the nanofibres are provided in Supplementary Materials (Fig. S1).

**Figure 3:**
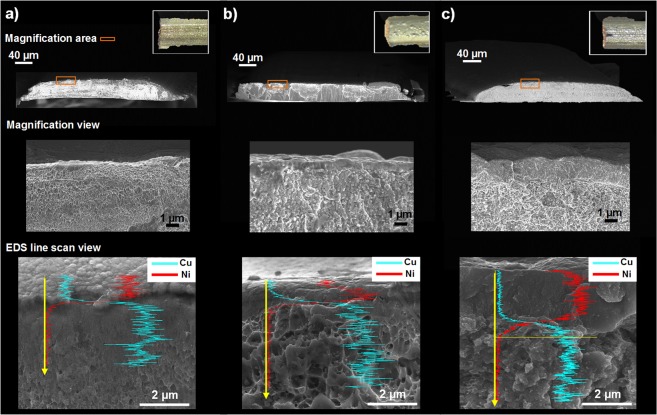
Optical top view (inset) and SEM cross section micrographs of printed copper-nickel bimetallic strips with a 3 hr (5 V vs Cu) copper deposition time and (**a**) 1 hr, (**b**) 3 hr and (**c**) 5 hr (2 V vs Ni) nickel deposition time along with accompanying EDS analysis.

Figure 3a–c shows optical images and SEM micrographs of the printed copper-nickel bimetallic strips at different deposition times as well as energy dispersive X-ray spectroscopy (EDS) line scans of the samples. In all cases, a lateral print head velocity of 0.4 mm.s^−1^ was used over a distance of 20 mm, with a 5 V vs Cu and 2 V vs Ni deposition potential for copper and nickel respectively. A base layer of copper was used in all cases with a 3 hr deposition time which produced samples with a thickness of approximately 45 μm after 216 passes which suggests a copper deposition layer resolution of approximately 200 nm. The morphological changes of the bimetallic strip can be observed with a nickel deposition time of 1 hr, 3 hrs and 5 hrs with the same print head velocity. This therefore represents 72, 216 and 360 passes respectively with a nickel layer thickness of approximately 600 nm, 1 μm and 2 μm and thus a layer resolution of approximately 5–8 nm. The finer layer height resolution of the nickel compared to the copper is due to the lower reaction current density due to the lower deposition potential and also slower deposition kinetics of nickel relative to copper. In all cases it can be seen from the SEM and the EDS line scans that a tightly bound and clear interface between the nickel and copper is formed, with the both metallic layers showing a polycrystalline, often nanocrystalline, morphology. The lower magnification images also highlight that in all cases the printed strips exhibit a convex shape which is due to the higher reaction current density in the centre of the deposition nozzle.

### Thermo-mechanical properties of bimetallic strips

**Figure 4:**
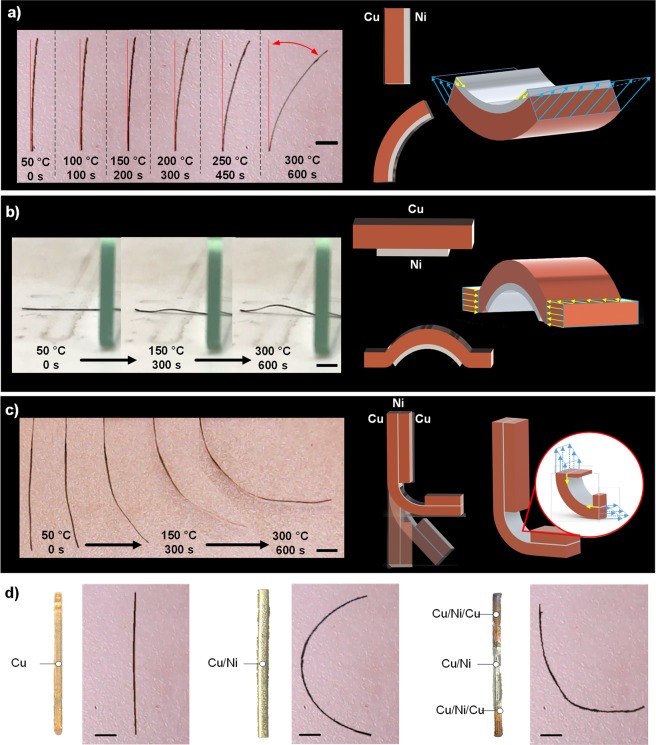
Thermo-mechanical response of different copper (3 h, 5 V vs Cu)-nickel Ni (5 hr, 2 V vs Ni) structures fabricated through a multi-nozzle ECAM approach. (**a**) Deformation of a Cu-Ni bimetallic strip with perpendicular heating. (**b**) Deformation of a Cu-Ni bimetallic strip with selective nickel deposition in the centre of the strip and heating with the strip flat against the heating bed. (**c**) Deformation of a Cu-Ni-Cu trilayer strip with Cu-Ni-Cu sandwich structures at both ends of the strip. (**d**) Optical images of samples programmed to exhibit the letters “ICL” at room temperature and 300 °C “ICL”. The scale bar is the same to all images in length of 2 mm.

In order to investigate the thermo-mechanical properties of the printed bimetallic strips, samples were placed onto a heated bed with one end fixed and the other free to move. An optical camera was placed over the samples to visualise the displacement as the temperature was increased from room temperature to 50 °C increments to 300 °C with images taken at each temperature once steady state conditions had been achieved. Measurements were repeated 3 times to confirm the performance. Figure 4 shows the deformation of the samples at different temperatures as well as with different copper-nickel layer configurations. It can be seen in all cases that as temperature is increased there is an observed mechanical deformation which is due to the difference in thermal expansion coefficient of copper (16 × 10^−6^ °C^−1^) and nickel (13 × 10^−6^ °C^−1^)^54^. Since the 2 metallic layers are tightly bound, a zero displacement boundary condition at the interface is assumed and thus internal stresses are generated in the layers. In this system, since the copper thermal expansion coefficient is higher than that of the nickel there is an observed curvature with a tensile stress is generated on the copper side and a compressive stress on the nickel side. For a bimetallic strip consisting of a 3 hr (~45 µm) copper and 5 hr (~2 µm) nickel deposition (Fig. 4a), a maximum deformation of 40° was measured at 300 °C. Here the bimetallic strip was positioned perpendicular to the heating bed. This deformation angle was measured by binarizing and fitting the acquired images to a circle to find the radius of curvature in MATLAB.

In Fig. 4b, the nickel region was limited to a central region of 6 mm with the same deposition conditions. When heating the sample with the nickel side down, it can be seen that a curvature in the copper-nickel region can be observed. In the pure copper regions at the ends of the strips this remains parallel to the heating bed, due to the lack of bending stresses and the force of the nickel-copper region applying a slight pressure on the copper region against the heated bed.

In Fig. 4c, a trilayer (Cu-Ni-Cu) configuration is shown which is similar to the Cu-Ni configuration (Fig. 4a) but with an additional copper layer printed on top of the nickel layer with an 8 mm gap in the centre. In this configuration it can be seen that the addition of the top copper layer balances the thermal stresses in the Cu-Ni bilayer region and thus prevents bending which enables “L-shaped” geometries to be achieved and thus programmable combinations of linear and curved structures. Using a combination of these designed structures, the letters “ICL” can be achieved after heating linear bimetallic sample to 300 °C (Fig. 4d).

Key design variables that affect the radius of curvature (*κ*) of the bimetallic strips include: the layer thicknesses, Young’s modulus and thermal expansion coefficient of the 2 layers as highlighted in Eq. 1 by Clyne and Gill^55^. Here *E*_*x*_, *h*_*x*_ and *α*_*x*_ are the Young’s modulus, thickness and thermal expansion coefficients of material *x* respectively, and Δ*T* represents the temperature difference between the reference temperature at zero strain and the operating temperature.

1 $$\kappa =\frac{6{E}_{1}{E}_{2}({h}_{1}+{h}_{2}){h}_{1}{h}_{2}({{\rm{\alpha }}}_{1}-{{\rm{\alpha }}}_{2}){\rm{\Delta }}T}{{E}_{1}^{2}{h}_{1}^{4}+4{E}_{1}{E}_{2}{h}_{1}^{3}{h}_{2}+6{E}_{1}{E}_{2}{h}_{1}^{2}{h}_{2}^{2}+4{E}_{1}{E}_{2}{h}_{2}^{3}{h}_{1}+{E}_{2}^{2}{h}_{2}^{4}}$$

**Figure 5:**
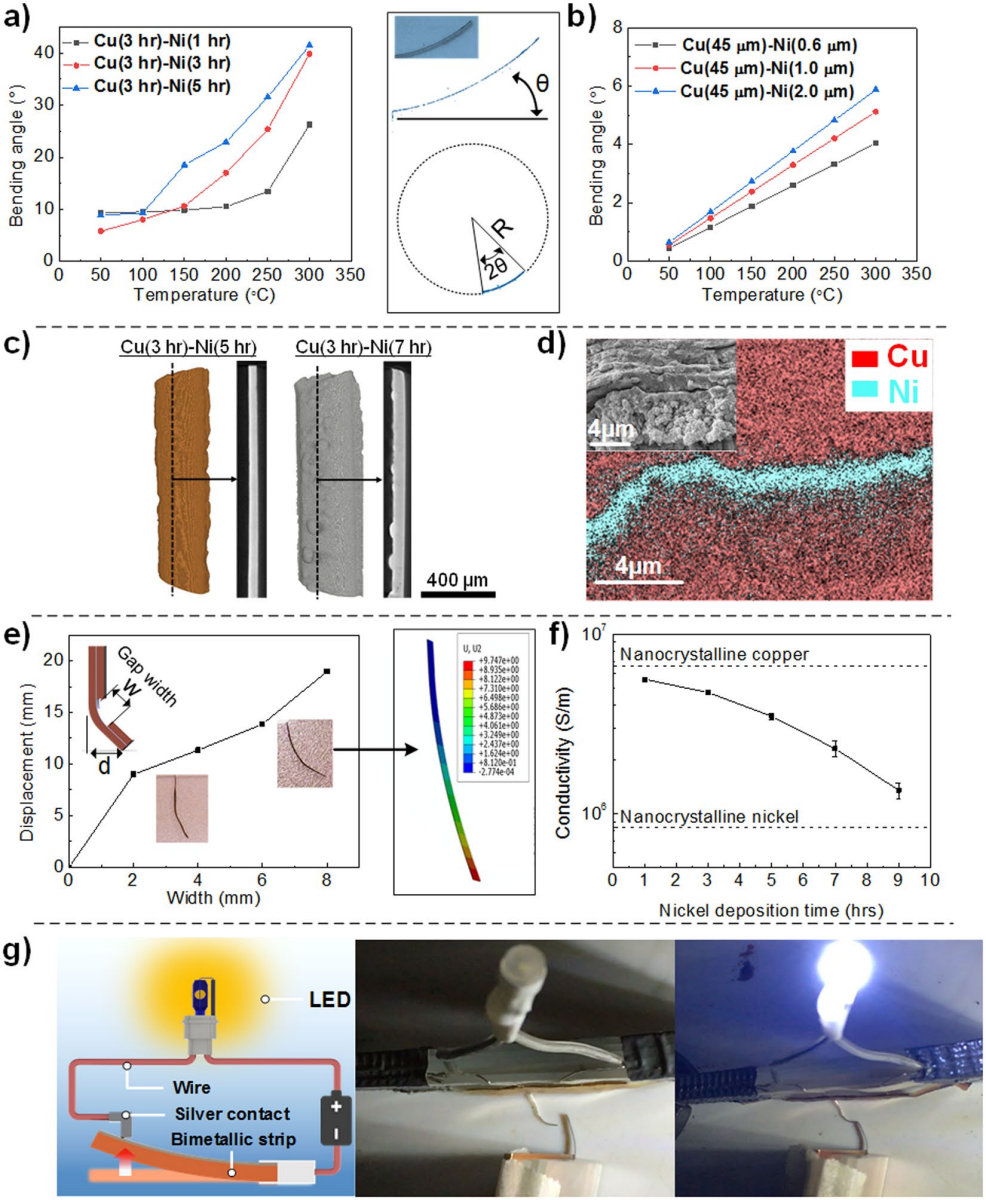
(**a**) Measured bending angles for different Cu-Ni bimetallic strips at different temperatures. (**b**) Theoretical bending angles of Cu-Ni bimetallic strips with ideallised geometries. (**c**) XCT reconstructions of Cu(3 hr)-Ni(1 hr) and Cu(3 hr)-Ni(5 hr) samples with reconstructed cross-section images. (**d**) SEM micrograph and EDS mapping of the Cu-Ni-Cu interface. (**e**) Displacement measurements of trilayer strips with varying gap width along with validated FEA simulations. (**f**) Electrical conductivity measurements of the bimetallic strips. (**g**) Schematic and photos of a simple electrical circuit actuated by the printed bimetalic strip.

Figure 5a, shows the bending angle of 20 mm Cu-Ni bimetallic strips with a base copper layer (5 V vs Cu, 3 hrs) and nickel layers with different thicknesses (2 V vs Ni for 1 hr, 3 hrs and 5 hrs) over a temperature range from room temperature to 300 °C. It can be seen that as the thickness of the nickel layer and temperature difference increases, the bending angle also increases non-linearly. When comparing this with the theoretical deflections based on Eqn. 1 (Fig. 5b), it can be seen that the same trend is observed, however the trend is linear and the absolute deflection angles are less. This can be explained by the fact that the deflection angles are highly sensitive to the layer thicknesses which from the SEM images in Fig. 3 is shown to vary due to the uneven current density in the deposition nozzle during printing causing the convex line cross section.

To investigate the 3D morphology of the printed structures, XCT was used (Fig. 5c). Here it can be seen that the convex cross section is reasonably maintained along the length of the sample in the copper phase with slight plating at the edges of the sample. When increasing the nickel plating duration, it can be seen that nodules of nickel are formed on the surface and that the coating is not even over the copper. This likely leads to the non-linear temperature-deflection behaviour (Fig. 5a**)**.

In the trilayer (Cu-Ni-Cu) configuration tight adhesion between the various layers is confirmed by a combination of SEM and EDS mapping (Fig. 5d**)**. An “L-shaped” deflection can be achieved (Fig. 4c). Figure 5e shows how this deflection can be controlled by changing the width between the Cu-Ni-Cu trilayers, with the deflection increasing as the gap distance increasing due to the larger region of exposed Cu-Ni. Finite element analysis (FEA) was then performed on the trilayer confirmation (Fig. 5e). Using the idealised geometry it was shown that the same general deformation was achieved.

Actuation of these printed bimetallic structures can also be achieved through ohmic heating since they are metallic. Figure 5f shows the electrical conductivity of the bimetallic strips, whereby all samples have a copper base layer fabricated with a 5 hr deposition time and 5 V vs Cu potential but varying nickel deposition times ranging from 1 hr to 9 hrs (2 V vs Ni). From the SEM images the nickel structure was polycrystalline with nano-sized grains. Thus, it is expected that the resistivity will be higher than course grained nickel as a result of electron scattering at grain boundaries^56^. The electrical conductivity of all bimetallic samples were found varying between 1.4 × 10^6^ S.m^−1^ and 5.5 × 10^6^ S.m^−1^ which was slightly lower than the electrical conductivity of the copper single line (6.86 × 10^6^ S.m^−1^) reported in Chen *et al*.^24^ but broadly inline with the conductivity of nanocrystalline copper reported by Lu *et al*.^57^ 5.4 × 10^6^ S.m^−1^. This is likely due to the lower electrical conductivity of nanocrystalline nickel (8.2 × 10^5^ S.m^−1^)^58^ compared to copper. Thus, as the deposition time of nickel increases, the electrical conductivity converges to that of Nickel.

Finally, to demonstrate the application of the printed bimetallic strips in high temperature environments, a simple circuit was built as shown in Fig. 5g. As the temperature increased to 300 °C, the Cu-Ni bimetallic strip bent and closed the circuit to power the LED. This highlights the potential application of this technique in fabricating structures that can sense the environment, thus opening the possibility for smarter 3D printed structures.

## Conclusions

In summary, a novel electrochemical 3D printer with 2 deposition nozzles was presented which is capable of printing temperature responsive multi-metal (copper and nickel) 4D structures. An electrospun nanofibre nib was used to provide sufficient back pressure to the hydraulic head exerted by the electrolyte, replacing the porous sponge material used in prior works by Chen *et al*.^24^. This new nib showed that it was capable of providing sufficient back pressure but with lower mass transport losses which thus increased the deposition speed by 34% for copper and 85% for nickel deposition. SEM imaging and EDS analysis showed that a tightly bound interface is formed between the copper and nickel layers, with printed lines exhibiting a convex cross section due to the higher reaction current density at the centre of the nozzle. Optical microscopy during heating of various printed samples including Cu-Ni and Cu-Ni-Cu structures demonstrated the ability of the technique in creating different movements. This was highlighted through the creation of structures such as the letters “ICL” and a simple temperature sensing circuit. Design variables that were investigated included the layer thicknesses and geometric arrangement of the different layers. It was shown that, for a constant copper base layer, the deflection of the bimetallic strip increased with increasing nickel layer thickness due to the different in thermal expansion coefficients of the 2 materials resulting in internal stresses in the printed structure. When comparing measured results with the analytical solution, differences were observed which has been attributed to the uneven deposition of nickel onto the copper which is confirmed by XCT analysis. The findings here thus present the first reported low cost multi-metal 3D printing approach for creating high temperature 4D structures which opens the possibility for creating more intelligent structures and sensors.

## Methods

### Electrochemical additive manufacturing

The low cost electrochemical multi-metal 4D printer was modified from a commercial FDM 3D printer (WER Me Creator) by replacing the heated extrusion head with two 10 mL polypropylene syringes (Metcal-7100LL1NPK) with 400 µm polypropylene nozzles (Metcal-922125-DHUV).

### Material preparation

Nickel deposition electrolytes were made through a mixture of NiSO_4_.6H_2_O 113 g.L^−1^, NiCl_2_.6H_2_O 30 g.L^−1^ and H_3_BO_3_ 23 g.L^−1^. 1 M copper sulphate electrolytes were prepared by mixing anhydrous copper (II) sulphate (12.5 g of CuSO_4_ ∙ 5H_2_O (≥99.99% pure – Sigma Aldrich)) 50 ml with deionized water.

### Electrochemical measurements

All deposition experiments and electrical conductivity measurements used a Metrohm Autolab PGSTA302N with a 4-electrode configuration. Electrical conductivity measurements were made on the same potentiostat with a 4-electrode configuration to isolate contact resistances under potentiostatic conditions.

### Microstructural characterization and imaging

A nanotom® s system (GE Sensing and Inspection Technologies GmbH, Wunstorf, Germany) was used for the XCT measurement. This was operated at an X-ray tube voltage and current of 120 kV and 100 µA respectively, with a tungsten-on-diamond target. The analysed samples were positioned between the X-ray source and detector to provide an effective pixel size of 0.6 µm. The 3D structures were reconstructed using 2,400 projection images captured over a 360° sample rotation and a cone-beam filtered back projection algorithm based on the Feldkamp-Davis-Kress (FDK) algorithm. Each projection image was acquired with an X-ray detector integration time of 1.5 s, with averaging and skip settings of 3 and 1 respectively, and an activated detector shift to minimise ring artefacts.

## Supplementary information

Multi-metal 4D printing with a desktop electrochemical 3D printer supplementary information
