# Supplementary material for: Multi-metal 4D printing with a desktop electrochemical 3D printer
Source: Sci Rep. 2019 Mar 8;9:3973. doi: 10.1038/s41598-019-40774-5 (PMC6408427; doi:10.1038/s41598-019-40774-5)
Supplement: Supplementary file 1 — Multi-metal 4D printing with a desktop electrochemical 3D printer supplementary information [file 41598_2019_40774_MOESM1_ESM.docx]

#### Supplementary Information

**Multi-metal 4D printing with a desktop electrochemical 3D printer**

**Xiaolong Chen1*, Xinhua Liu1+, Mengzheng Ouyang2, Jingyi Chen3, Oluwadamilola Taiwo2, Yuhua Xia2, Peter RN Childs1, Nigel P Brandon2 and Billy Wu2-**

1 Dyson School of Design Engineering, Imperial College London, UK

2 Department of Earth Science and Engineering, Imperial College London, UK

3 Department of Materials, Imperial College London, UK

Corresponding authors: *[x.chen15@imperial.ac.uk,](mailto:x.chen15@imperial.ac.uk) [+x.liu15@imperial.ac.uk,](mailto:%2Bx.liu15@imperial.ac.uk) [-billy.wu@imperial.ac.uk](mailto:-billy.wu@imperial.ac.uk)

## Electrospun nanofibre nib

### Nanofibre mats were formed through the electrospinning of polyacrylonitrile (PAN) dissolved in a dimethylformamide (DMF) solvent. As spun mats were then cut to 17 mm x 17 mm squares with a measured thickness of 50 µm. These mats were then rolled and placed into a tapered plastic nozzle with an end diameter of 400 µm. This is shown in **Figure S1**.


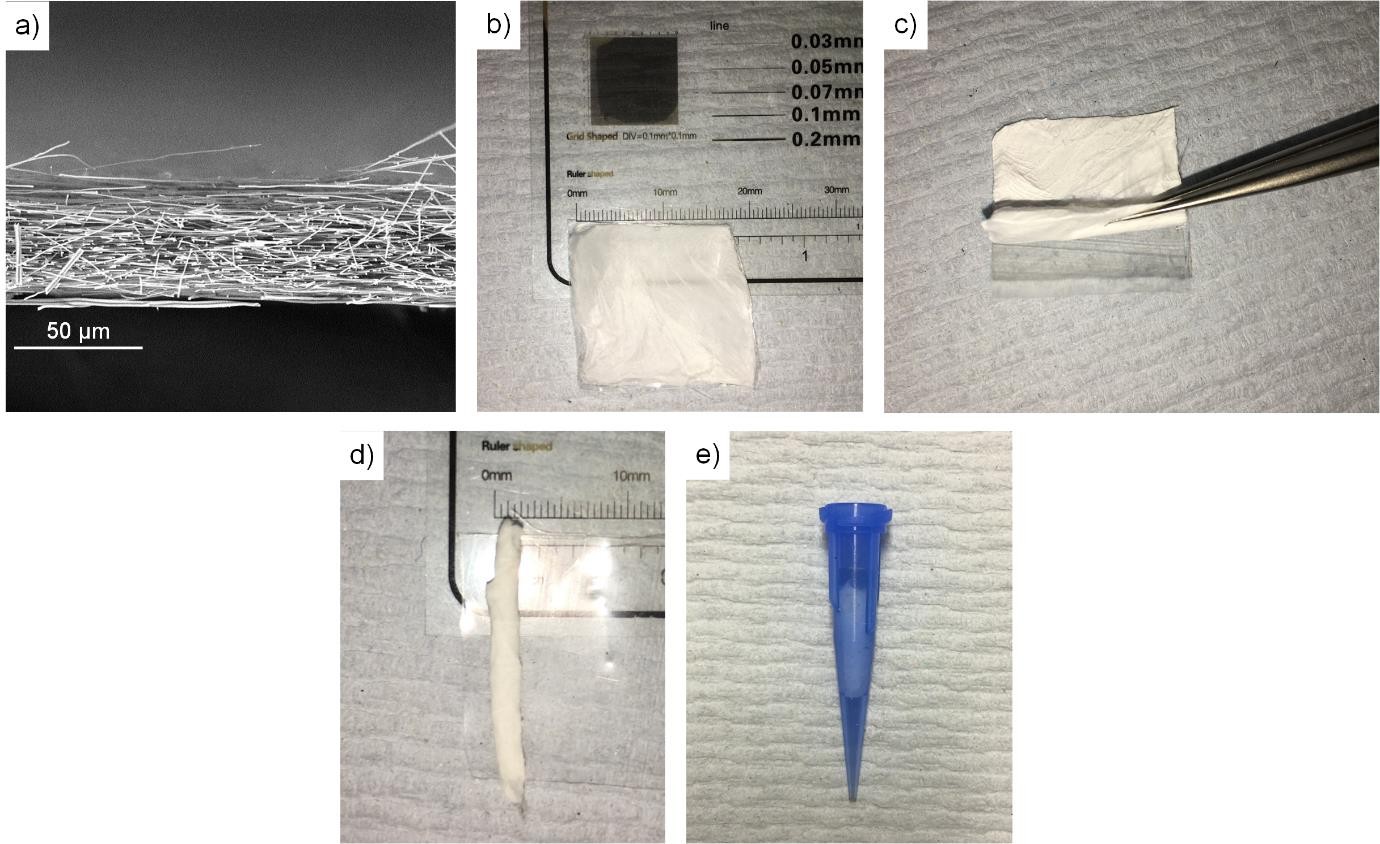


**Figure S1**: a) SEM image of the electrospun nanofibre mat. b) Photo of flat electrospun mat, c) rolling of the nanofibre mat, d) rolled nanofibre and e) nanofibre nib installed into the nozzle

## Optical images of printed strips

### **Figure S2** shows optical images of the printed samples which were taken on a Zeiss Stemi 508 optical microscope. All the samples were fabricated with a fixed deposition time of 5 hrs for the copper layer and deposition times of 1, 3 and 5 hrs for nickel.


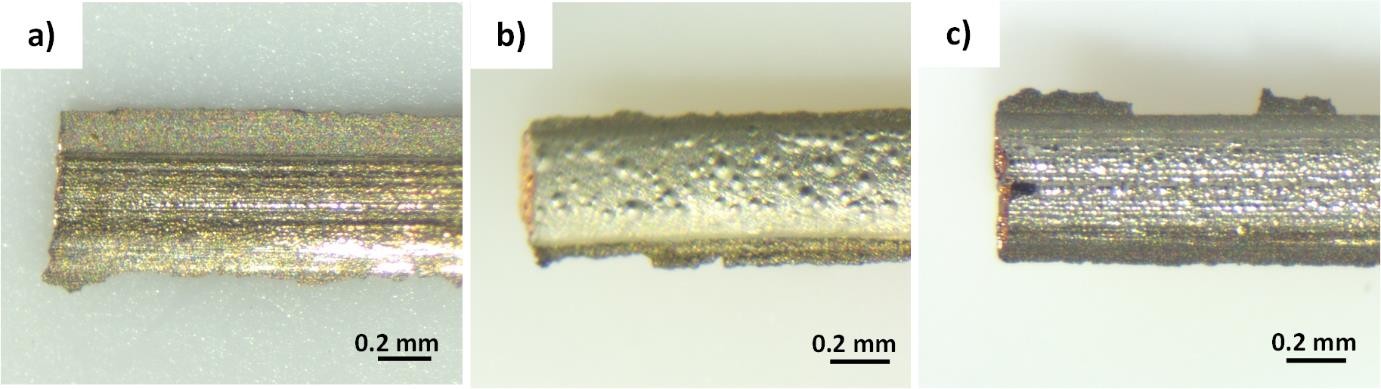


**Figure S2**: Optical images of the printed bimetallic strips with a 5 hr copper deposition and a) 1 hr, b) 3 hr and c) 5 hr nickel deposition time

## Detailed SEM and EDS micrographs for the Cu-Ni-Cu trilayer strip

### **Figure S3** shows a high magnification SEM image of the Cu-Ni-Cu trilayer with EDS mapping and spectra.


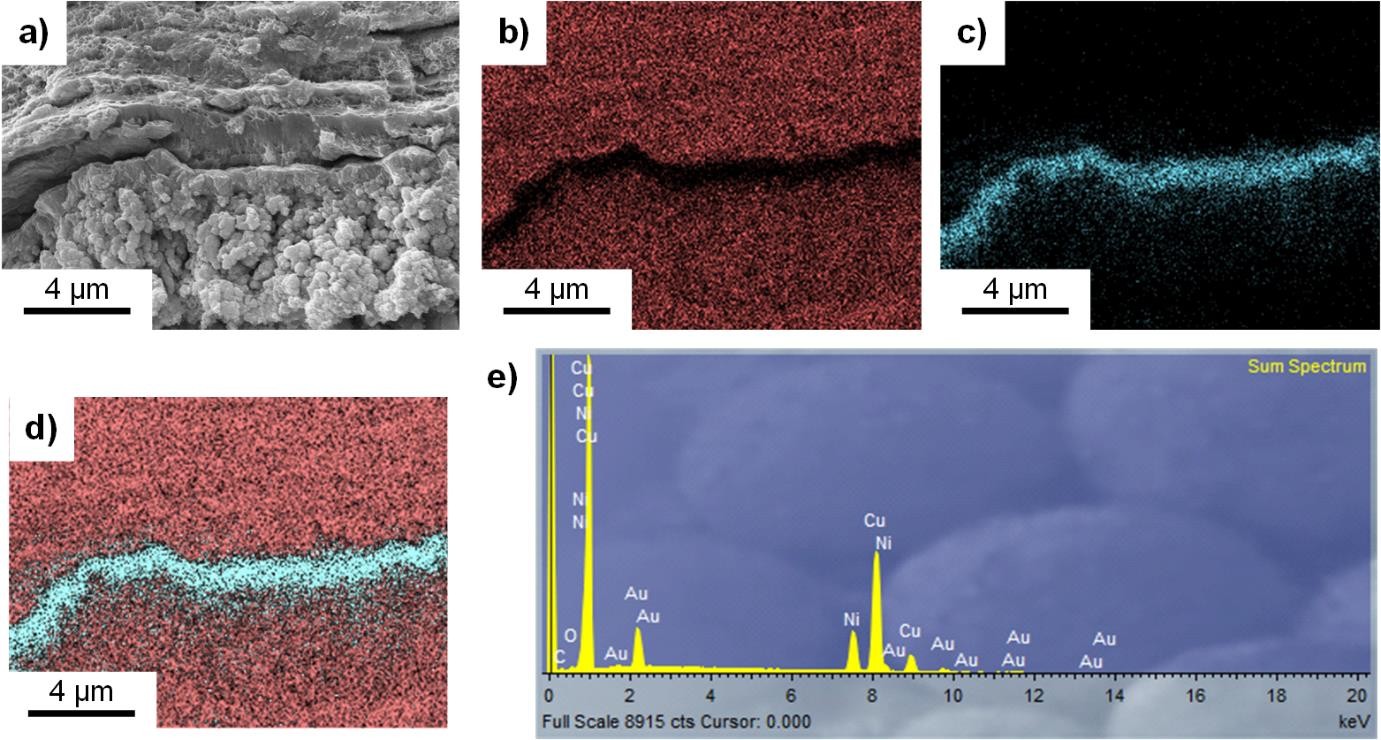


Figure S3: a) SEM micrograph of the cross section of a Cu-Ni-Cu trilayer strip. b) EDS mapping of Cu in the Cu-Ni-Cu trilayer strip, c) EDS mapping of nickel in the Cu-Ni-Cu trilayer strip, d) combined Cu and Ni map. e)

EDS spectra of the Cu-Ni-Cu strip
